# Adoptive Immunotherapy with Cl-IB-MECA-Treated CD8+ T Cells Reduces Melanoma Growth in Mice

**DOI:** 10.1371/journal.pone.0045401

**Published:** 2012-09-24

**Authors:** Antonella Montinaro, Giovanni Forte, Rosalinda Sorrentino, Antonio Luciano, Giuseppe Palma, Claudio Arra, Ian M. Adcock, Aldo Pinto, Silvana Morello

**Affiliations:** 1 Department of Pharmaceutical and Biomedical Sciences, University of Salerno, Salerno, Italy; 2 National Cancer Institute “G. Pascale” Naples, Naples, Italy; 3 NHLI, Imperial College of London, London, United Kingdom; Baylor College of Medicine, United States of America

## Abstract

Cl-IB-MECA is a selective A3 adenosine receptor agonist, which plays a crucial role in limiting tumor progression. In mice, Cl-IB-MECA administration enhances the anti-tumor T cell-mediated response. However, little is known about the activity of Cl-IB-MECA on CD8+ T cells. The aim of this study was to investigate the effect of ex vivo Cl-IB-MECA treatment of CD8+ T cells, adoptively transferred in melanoma-bearing mice. Adoptive transfer of Cl-IB-MECA-treated CD8+ T cells or a single administration of Cl-IB-MECA (20 ng/mouse) inhibited tumor growth compared with the control group and significantly improved mouse survival. This was associated with the release of Th1-type cytokines and a greater influx of mature Langerin+ dendritic cells (LCs) into the tumor microenvironment. CD8+ T cells treated with Cl-IB-MECA released TNF-α which plays a critical role in the therapeutic efficacy of these cells when injected to mice. Indeed, neutralization of TNF-α by a specific monoclonal Ab significantly blocked the anti-tumor activity of Cl-IB-MECA-treated T cells. This was due to the reduction in levels of cytotoxic cytokines and the presence of fewer LCs. In conclusion, these studies reveal that ex vivo treatment with Cl-IB-MECA improves CD8+ T cell adoptive immunotherapy for melanoma in a TNF-α-dependent manner.

## Introduction

Melanoma is the most aggressive skin tumor with high metastatic potential and only a 5% 5-year survival rate for patients with metastatic disease [1], [2]. The main feature of melanoma is the resistance to most chemotherapeutics [3]. Adoptive transfer of T cells is currently a promising anti-tumor therapy in patients with melanoma and many studies have generated functional T cells capable of mediating tumor regression *in vivo* [4]–[9].

Adenosine is a potent regulator of tumor immune-surveillance [10] and exerts its effect through four receptor subtypes: A1 and A3 receptors are Gi/o-coupled receptors that decrease intracellular cyclic AMP (cAMP) whilst A2a and A2b are Gs-coupled receptors that increase intracellular cAMP levels [11]. Increasing evidence shows that activation of different adenosine receptors might exhibit opposing outcomes on immune cell function: A2a and A2b receptors typically suppress cell responses, whilst A1 and A3 receptors promote cell activation [12]. A2a receptor activation critically impaired T cell function during activation, by reducing cytokine and chemokine production, which in turn facilitates tumor growth [10], [13]–[16]. However, little is known about the effect of adenosine A3 receptor agonists on T cells, which express the A3 receptor [17].

The A3 receptor plays a critical role in restricting tumor progression. Indeed, pharmacological activation of A3 receptor by its selective agonist Cl-IB-MECA arrests cell cycle progression of many cancerous cell lines and inhibits tumor growth in mice [10]. Furthermore, A3 receptor agonists enhance the anti-tumor activity of natural killer (NK) cells and increases serum levels of IL-12 in the mouse [18]. We have recently demonstrated that Cl-IB-MECA administration into mice can induce an efficient T cell response that could critically affect tumor growth [19].

In this study we sought to investigate whether Cl-IB-MECA-treated CD8+T cells are effective at controlling tumor growth. Here, we show that CD8+T cells, treated *in vitro* with Cl-IB-MECA, adoptively transferred into melanoma-bearing mice suppressed tumor growth. In addition, a single local injection of Cl-IB-MECA significantly reduced melanoma growth, facilitating a Th1-like and cytotoxic immune response in the tumor lesions. CD8+ T cells treated with Cl-IB-MECA secrete TNF-α which is crucial for the observed anti-tumor effects in mice.

## Materials and Methods

### Mice and Cell culture

C57Bl/6j and Athymic Nude-Foxn1^nu^ mice were purchased from Harlan Laboratories (Udine, Italy) and maintained in specific pathogen-free conditions in the Animal Facilities of the National Cancer Institute “G.Pascale” of Naples (Italy). This study was carried out in strict accordance with the recommendations in the Institutional animal care guidelines, Italian D.L. no. 116 of 27 January 1992 and European Communities Council Directive of 24 November 1986 (86/609/ECC). The ethics committee of Pharmaceutical and Biomedical Department of the University of Salerno approved this study.

B16-F10 mouse melanoma cell line was purchased from American Type Culture Collection (LGC Standards S.r.l., Milan, Italy) and cultured in DMEM supplemented with 10% FBS, L-Glutamine (2 mM), penicillin (100 U/ml) and streptomycin (100 U/ml) (Sigma-Aldrich, Milan Italy).

### Isolation and treatment of CD8+ T cells

CD8+ T cells were purified from the spleens of naïve C57Bl6j mice by magnetic separation using a CD8+ T cell isolation kit (negative selection, EasySep Stem Cell, Voden, Milan, Italy). Purity of CD8+ T cells was checked by flow cytometry after staining with a PE-conjugated anti-CD8 antibody (eBioscience, CA, USA) and was routinely around 90% (Figure S1). CD8+ T cells were cultured in RPMI 1640 enriched with 10% FBS and stimulated with Cl-IB-MECA (0.1 µg/ml; Tocris Cookson Ltd., London, UK) for 24 h or 72 h at a density of 10^6^ cells/ml. MRS 1191 (5 µM), an adenosine A3 receptor antagonist was also used. Cytokine production in supernatants was analyzed by ELISA and cells were stained with the following markers: CD27-FITC, CD25-PE, CD69-allophycocyanin and analyzed by FACS analysis. In some experiments CD8+ T cells were activated with Mouse T-Activator CD3/CD28 Dynabeads (Invitrogen, Milan, Italy), according to the manufacturer's instructions.

### Animal protocols

C57Bl6j mice (female at 6–10 weeks of age) were injected subcutaneously (s.c.) with 3×10^5^ B16 melanoma cells per mouse (0-day). Ten days later (10-day) mice were peritumorally (p.t.) administered once with Cl-IB-MECA (20 ng/mouse) or PBS (100 µl) and sacrificed 4 days later.

For the adoptive transfer of CD8+ T cells, tumor-bearing mice were injected p.t. with 1×10^6^ CD8+ T cells per mouse (in 100 µl PBS). Adoptively transferred CD8+ T cells were treated overnight with Cl-IB-MECA (0.1 µg/ml) or PBS, washed twice in PBS and immediately injected into melanoma-bearing mice. Tumor growth was monitored by measuring the perpendicular diameters by means of a calliper (Stainless Hardened, Ted Pella, Inc. CA, USA) and calculated by the formula 4/3 π×(long diameter/2)×(short diameter/2)^2^. For survival rate experiments, s.c. tumor volume was daily monitored and mice were be euthanized according to the animal care protocol when the tumor reached ∼2000 mm^3^ in volume. Melanoma tissues and proximal lymph nodes were isolated for further analyses.

In some experiments, a neutralizing monoclonal antibody (mAb) against TNF-α (mouse IgG Clone MP6-XT3, 10 µg/mouse; eBioscience, San Diego, CA, USA) [20] was injected i. p. every day, starting from day 10 when mice received Cl-IB-MECA alone or CD8+ T cells as described above. The anti-TNF-α mAb reduced levels of TNF-α detection by 95% compared with IgG (data nor shown).

### Flow Cytometry Analysis

Tumors and lymph nodes were harvested from mice after adoptive transfer and digested by collagenase A (1 U/ml) (Sigma-Aldrich, Milan, Italy). Samples were passed through 70-µm cell strainers and red blood cells were lysed. Cell suspensions were used for flow cytometric analyses (BD FacsCalibur, Milan, Italy). The following antibodies were used: CD8-PE, NK1.1-PE, CD3-PeCy5.5, CD11c-FITC, CD11b-PeCy5.5, F4/80-PE, CD4-FITC, CD207-PE or CD207- allophycocyanin, CD80-PE, MHC I-PeCy5.5 (eBioscience, San Diego, CA, USA). The stained cells were analysed by using Becton Dickinson FACScan flow cytometer.

### ELISA

TNF-α, IFN-γ and Granzyme B were detected in melanoma tissue homogenates and cell surnatants using a mouse specific ELISA kits (eBioscience, San Diego, CA, USA).

### Immunohistochemistry

For histological analysis, frozen tumor specimens were fixed with acetone, permeabilized with methanol, and stained with Hematoxilin and Eosin (H&E staining) according to standard procedures. Additional frozen sections were stained with Ki67 (Abcam, Cambridge, UK) or Granzyme B (Invitrogen, Milan, Italy) and detected with FITC-labeled anti-rabbit or FITC-labeled anti mouse secondary antibodies, respectively. In all staining experiments, isotype-matched IgG and omission of the primary antibody was used as negative controls. Tissue sections were stained for the presence of apoptosis using the TUNEL apoptosis kit (BioVision, CA, USA) according to the manufacturer's instructions. Melanoma sections were read in a blinded manner by two independent investigators. Labeled cells were counted per visual field and expressed as the number of TUNEL+ cells per mm^2^ ± SEM (n = 5 for each tissue) by means of Axioplan Imaging Programme (Carl Zeiss).

### Analysis of RNA

Total RNA from isolated CD8+ T cells was prepared using an RNASPIN MINI extraction kit according to the manufacturer's instructions (GE Healthcare, Milan, Italy). Reverse transcription was performed by using a first-strand cDNA synthesis kit (GE Healthcare) followed by PCR. Thermal cycling conditions were 5 min at 95°C, followed by 35 cycles of 45 sec at 94°C, 20 sec at 58°C, 30 sec at 72°C. The A3 receptor primer pairs were as follows:

Forward 5′- GTTCCGTGGTCAGTTTGGAT -3′

Reverse 5′- GCGCAAACAAGAAGAGAACC -3′.

### Statistical analysis

Results are expressed as mean ± SEM. All statistical differences were evaluated by either Student's *t* test or one way ANOVA as appropriate. To assess survival rate, the Kaplan-Mayer model was used, and comparison of survival between groups was performed using the log-rank test with Prism4 software (GraphPad Software, La Jolla, CA). P values less than 0.05 were considered statistically significant.

## Results

### Adoptively transferred CD8+ T cells treated with Cl-IB-MECA suppress melanoma growth

**Figure 1:**
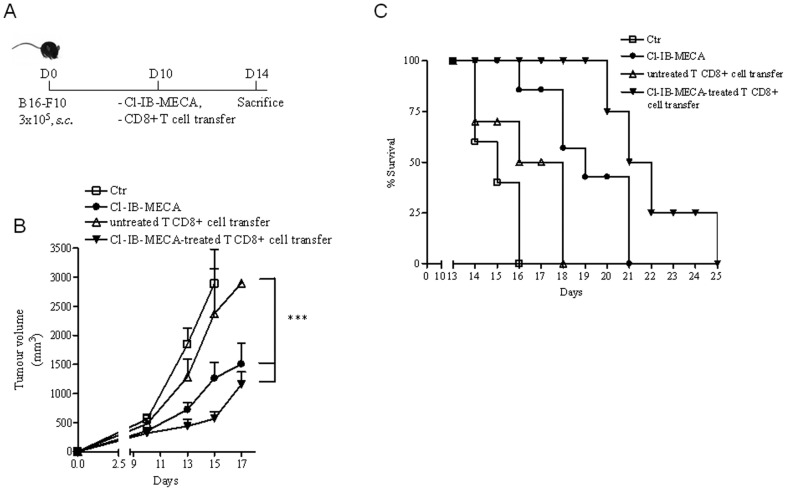
Adoptive transfer of CD8+ T cells cultured with Cl-IB-MECA mediates enhanced tumor suppression in melanoma-bearing mice. **A**) C57Bl6j mice were s.c. inoculated with 2.5×10^5^cells B16-F10 cells/mouse. 10 days after B16 injection, mice received a single p.t. injection of Cl-IB-MECA (20 ng/mouse) or 1×10^6^ CD8+ T cells/mouse treated with Cl-IB-MECA (0.1 µg/ml) or 1×10^6^ untreated CD8+ T cells/mouse or PBS (Ctr). **B**) Tumor volume (mm^3^) measured in control mice (Ctr, n = 11) and mice receiving a single dose of Cl-IB-MECA (n = 13) or adoptively transferred with Cl-IB-MECA-treated CD8+ T cells (n = 14) or with untreated CD8+ T cell (n = 10). **C**) Increased survival of melanoma-bearing mice receiving Cl-IB-MECA (p<0.05) or CD8+ T cell transfer cultured with Cl-IB-MECA (p<0.001) compared with control groups (n = 5/group). Data are from three independent experiments and represent mean ± SEM. Statistical differences were determined by one way ANOVA and Student's t test, as appropriate. ***p<0.001. Comparison of survival between groups was performed using long-rank test.

We assessed whether CD8+ T cells, treated *in vitro* with Cl-IB-MECA, have the capacity to control melanoma growth. CD8+ T cells, isolated from the spleen of naïve C57Bl6j mice, were treated overnight with Cl-IB-MECA (0.1 µg/ml) or PBS. Mice bearing B16 melanoma were adoptively transferred with Cl-IB-MECA- or PBS-treated CD8+ T cells (1×10^6^ cells/mouse) at day 10 after tumor implantation (Figure 1A). We found that adoptively transferred CD8+ T cells treated with Cl-IB-MECA significantly suppressed tumor growth compared with groups receiving T cells cultured without Cl-IB-MECA (untreated CD8+ T cell transfer) or controls (no adoptive transfer) (Figure 1B). The decrease in tumor growth was also verified by H&E staining (Figure S2A) and by Ki67 staining of melanoma sections (Figure S2B). The route of adoptive transfer of cells did not affect the ability of Cl-IB-MECA-treated cells to modulate tumor growth since a similar response to that described above was observed when cells were delivered by the i.v. route (Figure S3).

We also tested the anti-tumor activity of Cl-IB-MECA injected once into melanoma-bearing mice by peritumoral injection, which is an important route of administration to evaluate directly the effect of Cl-IB-MECA on tumor growth. We observed that mice receiving a single injection of Cl-IB-MECA (20 ng/mouse) showed a significant reduction in tumor growth compared with control mice (Figure 1B). In line with our previous data [19], this effect was associated with an increased number of tumor-infiltrating CD8+ T cells (Figure S4).

In addition, we adoptively transferred Cl-IB-MECA-cultured or control CD8+ T cells in melanoma-bearing mice and monitoring survival. Mice had to be euthanized according to the animal care protocol when tumor reached >∼2000 mm^3^ in volume. Mice injected with Cl-IB-MECA-treated CD8+ T cells showed a prolonged survival compared to mice injected with untreated CD8+ T cells or control groups (p<0.001). Specifically, 50% of control mice were alive at day 15, whereas 50% of mice adoptively transferred with Cl-IB-MECA-treated CD8+ T cells survived until day 21 (Figure 1C). Moreover, a single administration of Cl-IB-MECA alone was sufficient to improve survival (p<0.05) (50% of mice survived at day 19) (Figure 1C).

### *In vivo* effectiveness of Cl-IB-MECA-treated CD8+ T cells in melanoma

**Figure 2:**
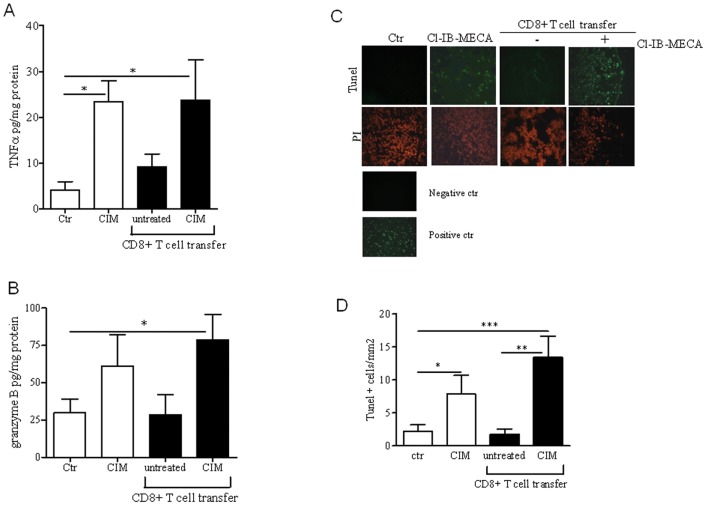
*In vivo* effectiveness of CD8+ T cell transfer after Cl-IB-MECA treatment is associated with increased apoptosis rate, granzyme B and TNF-α release into melanoma lesions. **A**) and **B**) TNF-α and granzyme B levels, respectively, detected into tissue homogenates from mice adoptively transferred with Cl-IB-MECA-stimulated CD8+ T cells or Cl-IB-MECA-treated mice. **C**) Representative pictures of melanoma cryosections Tunel stained (FITC) and stained with PI (red). Positive and negative controls are also provided. **D**) Quantitative analysis of Tunel+ cells detected in melanoma sections. Results are expressed as mean ± SEM (n = 5/group). Data are from two independent experiments and represent mean ± SEM, n = 6 in each experiment. Statistical difference was determined by one way ANOVA. *p<0.05, **p<0.01, ***p<0.001.

We then characterized the *in vivo* effectiveness of Cl-IB-MECA-treated CD8+ T cells in melanoma. TNF-α levels in melanoma tissue of mice adoptively transferred with Cl-IB-MECA-treated CD8+ T cells or mice injected once with Cl-IB-MECA were significantly increased compared with control groups (Figure 2A). Moreover, levels of granzyme B in melanoma tissue were elevated in mice adoptively transferred with Cl-IB-MECA-treated CD8+ T cells compared with those injected with untreated CD8+ T cells or PBS (Figure 2B).

We also analyzed apoptosis within the melanoma tissue. The number of TUNEL+ cells was increased in melanoma tissue harvested from mice adoptively transferred with Cl-IB-MECA-treated CD8+ T cells compared to that observed in untreated CD8+ cell transfer or controls (Figure 2C and D).

### Cl-IB-MECA-treated CD8+ T cells reduce tumor outgrowth in Nude mice

**Figure 3:**
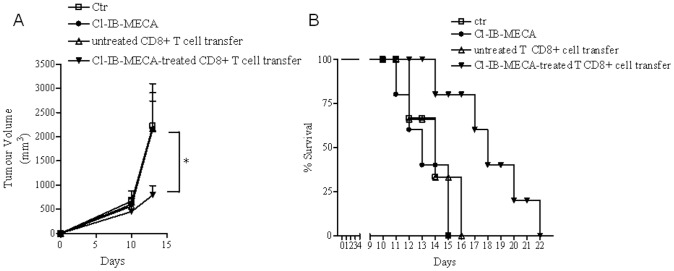
Tumor growth is inhibited in Nude mice adoptively transferred with CD8+ T cells stimulated with Cl-IB-MECA. **A**) Tumor volume (mm^3^) measured in melanoma-bearing Nude mice receiving a single dose of Cl-IB-MECA or adoptively transferred with Cl-IB-MECA-treated CD8+ T cells or with untreated CD8+ T cells. **B**) Increased survival of melanoma-bearing Nude mice receiving CD8+ T cell transfer, cultured with Cl-IB-MECA (p<0.01) compared with control groups and Cl-IB-MECA-treated mice (n = 5/group). Data represent mean ± SEM, n = 6 in each of two independent experiments. Statistical difference was determined by one way ANOVA. *p<0.05. Comparison of survival between groups was performed using long-rank test.

To examine the role of CD8+ T cells treated with Cl-IB-MECA in tumor immunotherapy, we used Nude mice, which lack of T cells, bearing 10-day B16 tumors. Nude mice that received CD8+ T cells treated with Cl-IB-MECA, as described above, had a significant reduction in tumor size compared with animals receiving untreated CD8+ T cells or control animals (no adaptive transfer) (Figure 3A). Notably, tumor growth in Nude mice receiving Cl-IB-MECA alone was similar to that observed in the control group (Figure 3A). Moreover, mice injected with Cl-IB-MECA-treated CD8+ T cells showed a prolonged survival compared to mice injected with untreated CD8+ T cells or PBS or Cl-IB-MECA (p<0.01) (Figure 3B). H&E staining and Ki67 staining were also performed in melanoma sections harvested from melanoma-bearing Nude mice (Figure S2C and D, respectively).

These data suggest that Cl-IB-MECA-treated CD8+T cells are able to control tumor growth when adoptively transferred into melanoma-bearing mice and further support the concept that the anti-tumor activity of Cl-IB-MECA is mediated by CD8+ T cells.

### Cl-IB-MECA modulates CD8+T cell function *in vitro*

**Figure 4:**
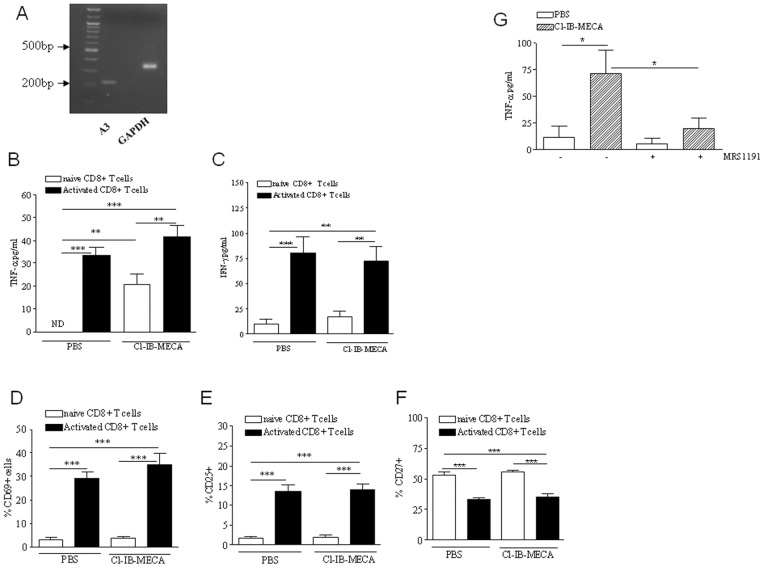
Cl-IB-MECA stimulation of CD8+ T cells *in vitro* favors TNF-α production. **A**) A3 adenosine receptor expression by CD8+ T cells. **B**) TNF-α levels in the supernatants of naïve (white bars) and activated (black bars) CD8+ T cells after Cl-IB-MECA treatment measured at 24 h by ELISA. **C**) INF-γ production measured in the supernatants of naïve (white bars) and activated (black bars) CD8+ T cells stimulated or not with Cl-IB-MECA (0.1 µg/ml) and measured at 72 h by means of ELISA. . **D**), **E**) and **F**) percentage of CD69+ cells, CD25+ cells or CD27+ cells, respectively, after Cl-IB-MECA treatment. **G**) MRS1191 blocked the stimulatory effect of Cl-IB-MECA on TNF-α release. Data are from two independent experiments and represent mean ± SEM, n = 8 for each experiment. Statistical difference was determined by Student's *t* test. *p<0.05.

The A3 receptor is expressed on T cells and its stimulation typically activates cell responses as a consequence of reduced intracellular cAMP levels [10], [12]. Figure 4A shows that CD8+ T cells expressed mRNA coding for the A3 adenosine receptor. Our observation that treatment of CD8+ T cells with Cl-IB-MECA significantly improved the efficacy of these cells when adoptively transferred into melanoma-bearing mice, led us to further investigate whether Cl-IB-MECA may affect the phenotypic and functional characteristics of CD8+ T cells. In both resting and activated conditions, Cl-IB-MECA-treated CD8+ T cells secrete TNF-α (Figure 4B, white bars) and little IFN-γ (Figure 4C, white bars). These results were confirmed by intracellular staining of TNF-α. Indeed, 4 h after Cl-IB-MECA stimulation the percentage of CD8+TNF-α+ cells increased compared with control cells (1.75±0.05 vs 16.3±0.96%). In vitro, Cl-IB-MECA treatment did not affect the activation status of CD8+ T cells. The number of CD8+ CD69+ T cells, CD8+CD25+ T cells and CD8+CD27+ T cells did not change after Cl-IB-MECA treatment compared with PBS (Figure 4D, E and F respectively, white bars). The same experiments were also performed in CD8+ T cells activated with anti-CD3/CD28. We found no change in the expression of CD69, CD25 and CD27 (Figure 4D, E and F respectively, black bars) nor increased levels of TNF-α and IFN-γ in activated CD8+ T cells after Cl-IB-MECA stimulation. Indeed, levels of TNF-α and IFN-γ were similar to those observed in control cells (Figure 4B and C, black bars).

To verify that the A3 adenosine receptor mediated the effect of Cl-IB-MECA on TNF-α release in CD8+ T cells, we treated cells with the A3 receptor antagonist MRS1191 (5 µM) before the addition of Cl-IB-MECA or PBS. MRS1191 completely blocked the effect of Cl-IB-MECA on TNF-α production (Figure 4G).

Together, these results suggest that Cl-IB-MECA failed to directly activate CD8+ T cells, but favors the production of TNF-α in an A3-dependent manner. Accordingly, we also found that splenocytes isolated from melanoma-bearing mice treated with Cl-IB-MECA released increased amounts of TNF-α compared with splenocytes from control animals (data not shown).

### The anti-tumor activity of Cl-IB-MECA-treated CD8+T cells is dependent on TNF-α

**Figure 5:**
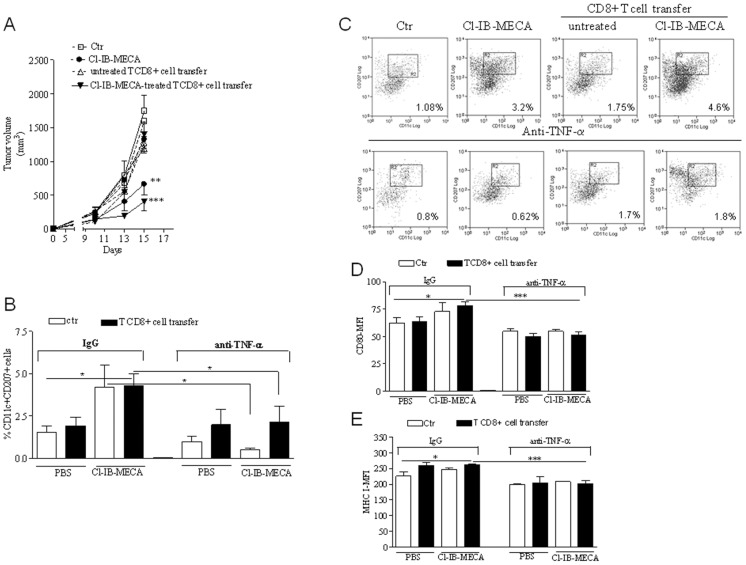
Neutralization of TNF-α abrogated the anti-tumor effect of Cl-IB-MECA-treated CD8+ T cells. **A**) Tumor volume (mm3) in mice receiving anti-TNF-α mAb (dashed lines) or isotype IgG control (continuous lines) and injected with Cl-IB-MECA or PBS or CD8+ T cells treated or not with Cl-IB-MECA. **B**) Percentage of CD11c+CD207 high cells in the tissue of mice described above. **C**) Representative dot plot is shown. **D**) and **E**) Expression of CD80 and MHC I, respectively, on CD11c+CD207 high cells in the tissue of mice described above. Data are from two independent experiments and represent mean ± SEM, n = 9 for each experiment. Statistical difference was determined by one way ANOVA. *p<0.05, **p<0.01, ***p<0.001.

To evaluate the role of TNF-α derived from Cl-IB-MECA-treated CD8+ T cells, CD8+ T cells were transferred into melanoma bearing mice receiving a neutralizing monoclonal antibody (mAb) against TNF-α. For this purpose, mice were injected with 10 µg/mouse of mAb anti-TNF-α or IgG control (mouse IgG) every day starting from day 10 after tumor cell implantation. Mice were administered with Cl-IB-MECA once or adoptively transferred with untreated CD8+ T cells or Cl-IB-MECA-treated CD8+ T cells as described above. Tumor growth in mice treated with anti-TNF-α mAb (dashed lines) was compared with that measured in mice treated with the IgG control (continuous lines) (Figure 5A). The administration of the anti-TNF-α mAb did not alter the tumor growth in melanoma-bearing control mice (Figure 5A). The capacity to control tumor growth by transferred Cl-IB-MECA-treated T cells was significantly affected in mice receiving the TNF-α mAb (Figure 5A). Similarly, the anti-tumor effect of Cl-IB-MECA alone was abrogated in mice injected with TNF-α mAb (Figure 5A). These data correlated with a reduction of granzyme B within tumor tissue whereas no reduction in granzyme B levels were seen in IgG-treated melanoma-bearing mice (data not shown). These results indicate that TNF-α within the tumor tissue could be critical for melanoma cell destruction. Analysis of leukocytes in the melanoma tissue revealed that the percentage of CD11c+ Langerin (CD207) high dendritic cells (LCs, Langerhans cells) significantly increased in melanoma tissue of mice adoptively transferred with Cl-IB-MECA-treated CD8+ T cells (Figure 5B and C; black bar) compared with control mice (Figure 5B and C; white bar). Moreover, the activation markers CD80 (66.3±3.2 versus 78.0±3.6; p<0.05; Figure 5D) and MHC class I (236.4±14.0 versus 261.8±2.0; p<0.05; Figure 5E) were significantly increased on LCs of mice adoptively transferred with Cl-IB-MECA-treated CD8+ T cells. Thus, transfer of Cl-IB-MECA-treated CD8+ T cells which are able to produce TNF-α, was associated with a significant recruitment of LCs into the tumor tissue.

In mice adoptively transferred with Cl-IB-MECA-treated CD8+ T cells the presence of CD11c+CD207+ cells (Figure 5B and C) and the expression of the activation markers CD80 (78.0±3.6 versus 51.6±2.2; p<0.001; Figure 5D) and MHC I (261.8±2.0 versus 201.8±9.4; p<0.001; Figure 5E) were significantly reduced after TNF-α neutralization.

Altogether, these results suggest that TNF-α released from Cl-IB-MECA-treated CD8+ T cells or after Cl-IB-MECA administration induced LCs activation/influx in the melanoma tissue, which could, in turn, facilitate the T cell responses *in vivo*.

## Discussion

In this study we show that Cl-IB-MECA -treated CD8+ T cells adoptively transferred into tumor-bearing mice, control melanoma growth. Cl-IB-MECA is a selective agonist of the A3 adenosine receptor, which plays a critical role in limiting tumor progression [10]. A large number of studies have demonstrated the therapeutic potential of Cl-IB-MECA as an anti-cancer agent due to its ability to inhibit tumor cell proliferation both *in vitro* and *in vivo* [21]. We have previously demonstrated that Cl-IB-MECA did not affect the proliferation rate of B16-F10 cells *in vitro*, but significantly reduced melanoma growth in mice, favoring a Th1-like immune response in the tumor microenvironment [19]. Here we show that a single injection of Cl-IB-MECA efficiently suppress tumor growth in mice. The anti-tumor activity of Cl-IB-MECA is lost in mice lacking T cells, further supporting its high potential to positively affect the T cell-mediated immune response against cancer cells. This effect was accompanied by increased levels of Th1-like and cytotoxic cytokines in the tumor milieu, which are critical for anti-tumor activity in the host [22], [23]. The data suggests an indirect effect on IFN-γ expression, perhaps by priming CD8+ T cells, since Cl-IB-MECA does not affect IFN-γ production directly.

Our results show, for the first time, that Cl-IB-MECA can influence CD8+ T cells function. Upon Cl-IB-MECA treatment, these cells significantly reduced tumor growth and improved survival when adoptively transferred into melanoma-bearing hosts. The reduction of tumor growth was associated with higher TNF-α and granzyme B production, which are both known to induce programmed cell death [24]. The superior anti-tumor activity of these cells was also shown in melanoma-bearing Nude mice.

Numerous studies have programmed CD8+ T cells to release IFN-γ *in vitro* as this is associated with an effector phenotype [8], [25], [26]. These criteria are generally sufficient to predict anti-tumor activity *in vivo* on adoptive transfer, although the acquisition of full effector function *in vitro* impairs *in vivo* anti-tumor efficacy [27]. It has also been reported that the effectiveness of adoptively transferred T cells *in vivo* can be independent of IFN-γ production and is correlated with other effector cell functions, such as production of cytotoxic mediators including TNF-α, perforin and FasL [28]–[32]. Under our experimental conditions, CD8+ T cells stimulated *in vitro* with Cl-IB-MECA secrete TNF-α, but not IFN-γ. Our data, therefore, supports the concept of an indirect effect of Cl-IB-MECA on CD8+ T cell IFN-γ-production.

T cells may produce toxic cytokines, such as TNF-α, which are able to induce apoptosis [29], [30], [32]. However, in addition to its well-defined role in apoptosis, TNF-α can critically affect the immune response at tumor sites. TNF-α is indeed critical for the anti-tumor T cell immunity in mice and is required for the optimal functional T cell response to tumors [33]. The effects of adoptively transferred Cl-IB-MECA-treated CD8+T cells *in vivo* could, therefore, be due to a locally released cytokines, such as TNF-α, in the tumor microenvironment. TNF-α could induce a local response which favors the recruitment/activation of other immune cells, such as dendritic cells [33], [35]. Therefore, the positive outcome that occurs *in vivo* may be achieved only in the tumor context.

Mice adoptively transferred with Cl-IB-MECA-treated CD8+ T cells showed a higher presence of mature LCs that could facilitate the T cell response [34].Transfer of Cl-IB-MECA-treated CD8+ T cells in mice receiving TNF-α mAb led to a reduced activation/influx of mature LCs within tumor tissue, which correlated with an impaired activity of CD8+ T cells to control tumor growth. Depletion of CD11c+ CD207 high cells in future studies may resolve whether the influx LC cells in response to Cl-IB-MECA is sufficient for its anti-tumour effect or whether the local release of TNFα by T-cells has additional effects. Taken together our *in vivo* evidence demonstrates the critical role of TNF-α in the Cl-IB-MECA-treated CD8+ T cell-induced immune response within the tumor lesion. However, our data cannot rule out the possibility that Cl-IB-MECA treatment could also influence the CD8+T cell activity in an antigen-dependent manner. This possibility using gp100 TCR transgenic mice (pmel) as donors should be tested in future studies.

In conclusion, our study demonstrates that adoptive transfer of Cl-IB-MECA-treated T cells reduced melanoma growth in mice and prolonged survival time. It is also noteworthy that a single administration of Cl-IB-MECA may augment the protective immunity in the melanoma, by enhancing tumor infiltration of CD8+ T cells, which, in turn, are driven to induce a Th1-like and cytotoxic immune microenvironment. The enhancement of the therapeutic efficacy of CD8+ T cells by Cl-IB-MECA further supports its utility in cancer immunotherapy.

## Supporting Information

Figure S1 **CD8+ T cell enrichment from naïve spleen.** CD8+ T cells were negatively selected from spleen of naïve C57Bl6j mice. Purity of CD8+ T cells was checked by flow cytometry analysis after staining with a PE-conjugated anti-CD8 antibody and was routinely around 90%. Cells were gated as CD3+CD8+ cells.
(TIF)

Figure S2 **A single injection of Cl-IB-MECA and Cl-IB-MECA-treated CD8+ T cell transfer suppress melanoma growth.** A) and B) H&E staining and Ki67 staining of melanoma-bearing C57Bl6i mice, respectively. C) and D) H&E staining and Ki67 staining of melanoma-bearing Nude mice, respectively. (Magnification: 20×).
(TIF)

Figure S3 **Cl-IB-MECA-treated CD8+ T cells reduce melanoma growth.** Cells were delivered by the i.v. route. Data are from two independent experiments and represent mean ± SEM, n = 6. Statistical difference was determined by one way ANOVA. *p<0.05.
(TIF)

Figure S4 **Cl-IB-MECA administration enhances the presence of CD8+T cells in the tissue.** Percentage of CD3+CD8+ T cells in tumor tissue of mice receiving a single injection of Cl-IB-MECA. Representative dot plot is shown on the right of the graph.
(TIF)
