# Supplementary figures and images for: Adoptive Immunotherapy with Cl-IB-MECA-Treated CD8+ T Cells Reduces Melanoma Growth in Mice
Source: PLoS One. 2012 Sep 24;7(9):e45401. doi: 10.1371/journal.pone.0045401 (PMC3454429; doi:10.1371/journal.pone.0045401)

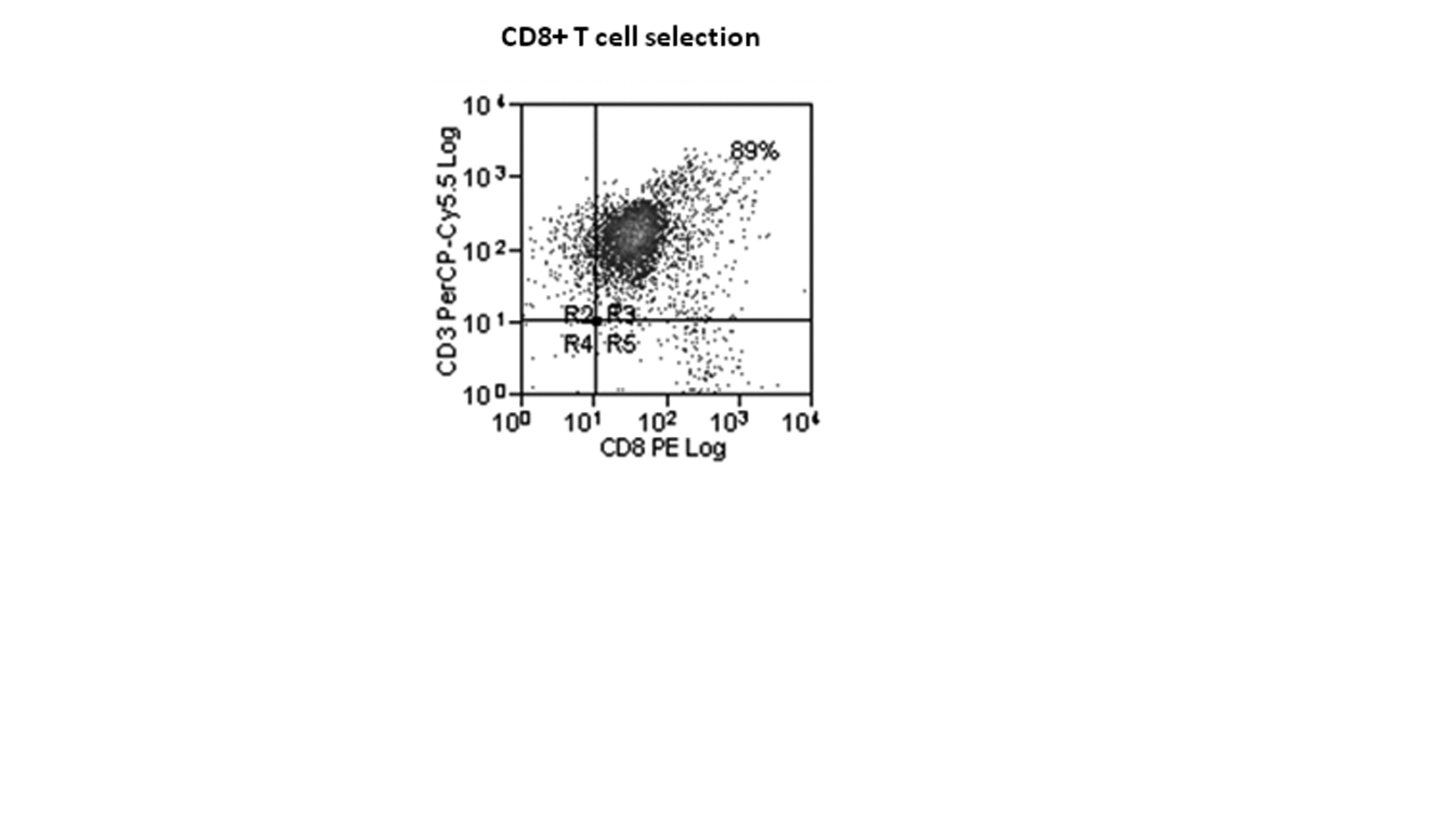

Supplement: Figure S1 — CD8+ T cell enrichment from naïve spleen. CD8+ T cells were negatively selected from spleen of naïve C57Bl6j mice. Purity of CD8+ T cells was checked by flow cytometry analysis after staining with a PE-conjugated anti-CD8 antibody and was routinely around 90%. Cells were gated as CD3+CD8+ cells. (TIF) [file pone.0045401.s001.tif]

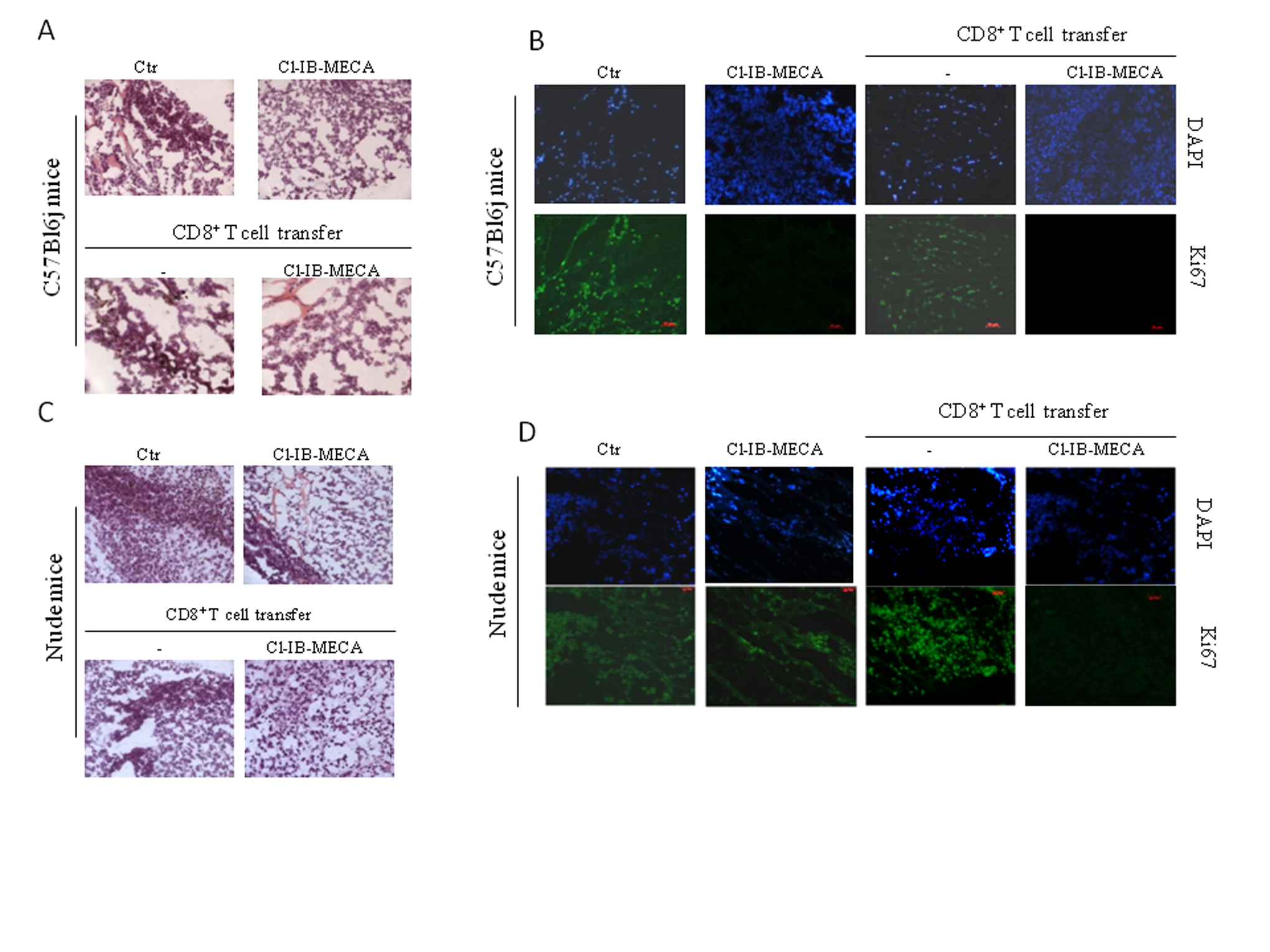

Supplement: Figure S2 — A single injection of Cl-IB-MECA and Cl-IB-MECA-treated CD8+ T cell transfer suppress melanoma growth. A) and B) H&E staining and Ki67 staining of melanoma-bearing C57Bl6i mice, respectively. C) and D) H&E staining and Ki67 staining of melanoma-bearing Nude mice, respectively. (Magnification: 20×). (TIF) [file pone.0045401.s002.tif]

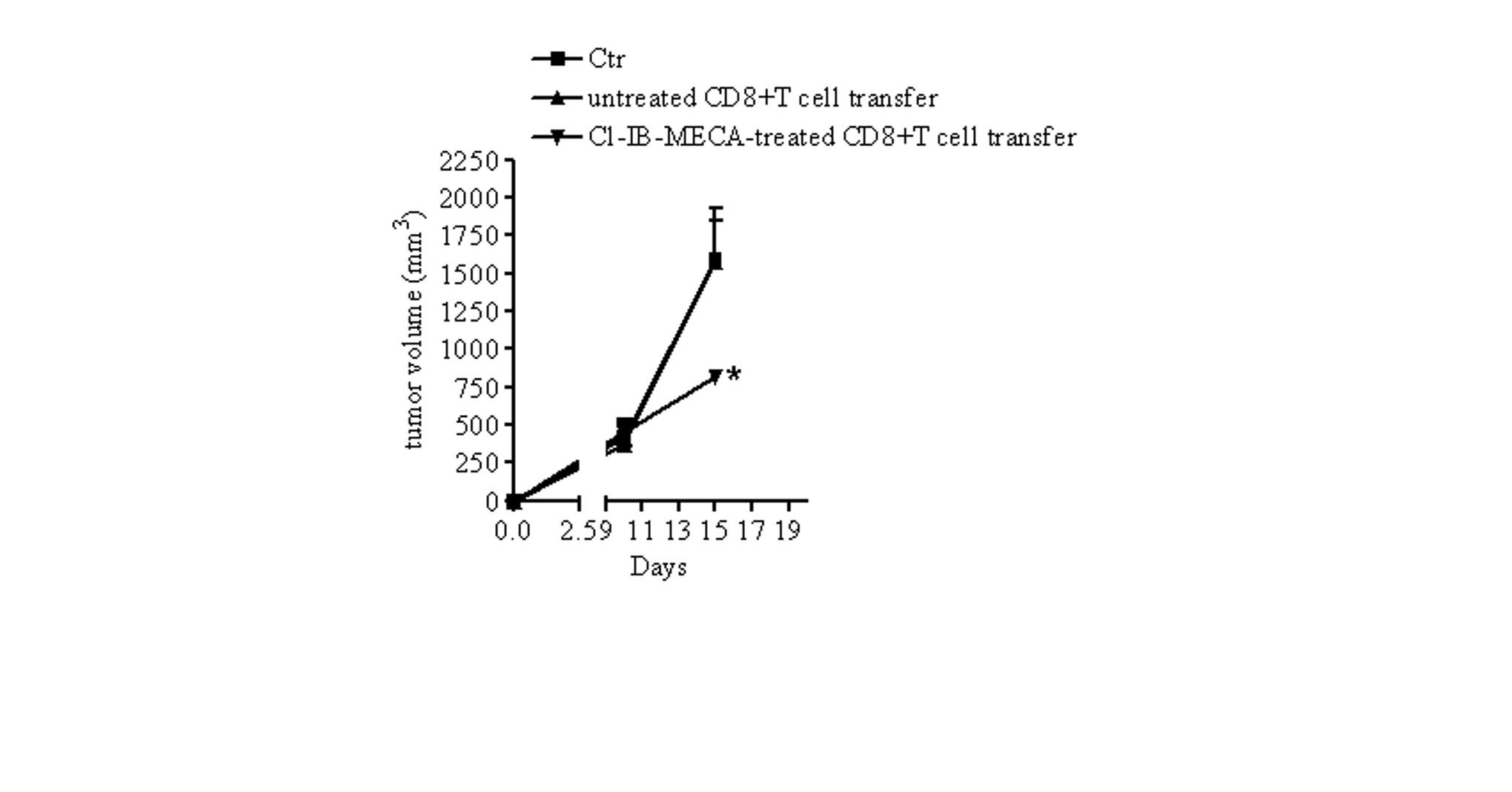

Supplement: Figure S3 — Cl-IB-MECA-treated CD8+ T cells reduce melanoma growth. Cells were delivered by the i.v. route. Data are from two independent experiments and represent mean ± SEM, n = 6. Statistical difference was determined by one way ANOVA. *p<0.05. (TIF) [file pone.0045401.s003.tif]

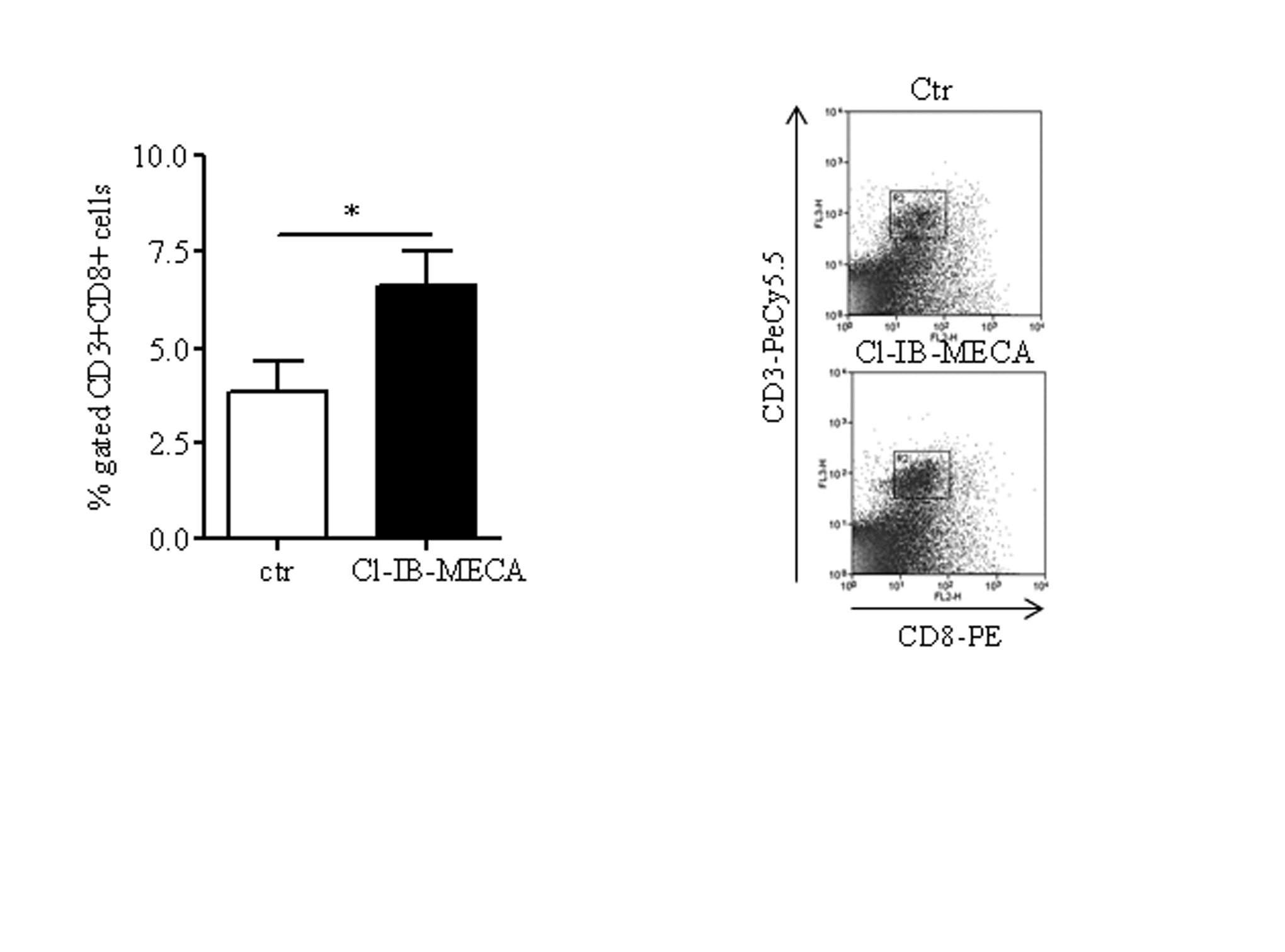

Supplement: Figure S4 — Cl-IB-MECA administration enhances the presence of CD8+T cells in the tissue. Percentage of CD3+CD8+ T cells in tumor tissue of mice receiving a single injection of Cl-IB-MECA. Representative dot plot is shown on the right of the graph. (TIF) [file pone.0045401.s004.tif]
